# Seasonal Changes of Thyroid Function Parameters in Women of Reproductive Age Between 2012 and 2018: A Retrospective, Observational, Single-Center Study

**DOI:** 10.3389/fendo.2021.719225

**Published:** 2021-09-02

**Authors:** Jinrong Fu, Guofeng Zhang, Pei Xu, Rui Guo, Jiarong Li, Haixia Guan, Yushu Li

**Affiliations:** ^1^Department of Endocrinology and Metabolism, Institute of Endocrinology, National Health Commission (NHC) Key Laboratory of Diagnosis and Treatment of Thyroid Diseases, The First Affiliated Hospital of China Medical University, Shenyang, China; ^2^Department of Endocrinology, Guangdong Provincial People’s Hospital, Guangdong Academy of Medical Sciences, Guangzhou, China; ^3^The Second School of Clinical Medicine, Southern Medical University, Guangzhou, China

**Keywords:** thyrotropin, thyroid hormones, seasonal changes, reproductive health, big data

## Abstract

**Background:**

Thyroid function can be influenced by external stimuli such as light and temperature. However, it is currently unknown whether there is seasonal variation of thyroid function in women of reproductive age. Adequate thyroid function in reproductive-aged women is necessary for optimal fetal–maternal outcomes. Therefore, this study aims to evaluate the seasonal changes in levels of thyrotropin (TSH), free triiodothyronine (FT_3_), free thyroxine (FT_4_), and TSH index (TSHI) in women of reproductive age.

**Methods:**

A large retrospective study was conducted that included women aged 20–49 years who visited our outpatient or checkup center between 2012 and 2018. Thyroid function was measured using the automated immunochemiluminescent assay kit. Subjects with overt thyroid dysfunction, pregnancy, thyroid disease, cancer, and severe infectious or psychological disease were excluded. Seasonal differences of thyroid function were analyzed using the Kruskal–Wallis test or the analysis of means with transformed ranks. Spearman’s correlation was performed to evaluate the association between thyroid function parameters and age. A subset of 181 subjects was included in the longitudinal analyses. Differences in thyroid function between summer and winter were analyzed using the Wilcoxon signed-rank test.

**Results:**

A total of 48,990 women with a median age of 39 years were included. The prevalence of subclinical hypothyroidism was lower in summer but higher in winter (5.6% *vs.* 7.0%, p < 0.05). The TSH, FT_3_, and FT_4_ levels and TSHI reached a peak in winter, while they declined to trough in summer. The TSH concentrations (r = 0.044, p < 0.001) and TSHI (r = 0.025, p < 0.001) were positively correlated with age, whereas FT_3_ (r = -0.073, p < 0.001) and FT_4_ (r = -0.059, p < 0.001) were negatively correlated with age. The associations of thyroid parameters with age were similar between subjects with positive thyroid peroxidase antibody (TPOAb) and those with negative TPOAb. In the matched longitudinal analysis of 181 subjects, no differences were detected in the thyroid parameters between summer and winter.

**Conclusions:**

This retrospective single-center study showed that thyroid hormone levels and central sensitivity to thyroid hormones are influenced by age and seasonal fluctuations among women of reproductive age, while their impact on reproductive health remains to be elucidated in future studies.

## Introduction

Most organisms have altered physiology in adaptation to environmental stimuli such as temperature, light, and rainfall (1). These changes in physiology may manifest as seasonal variations in metabolic, reproductive, and psychological systems (2–4). Thyroid hormones are key regulators of these systems. Their levels are tightly regulated by the hypothalamus–pituitary–thyroid (HPT) axis. The semiannual seasonal pattern of thyrotropin (TSH) levels has been well established and is presumed to be a promoter of seasonality in human physiology (5).

However, there are uncertainties in the generalizability of seasonal thyroid function variations in different population subgroups (6, 7). The fitness of women during the periconception period has been increasingly recognized as an important contributor in improving fetal–maternal outcomes. Maternal thyroid dysfunction before and during pregnancy has been associated with various adverse outcomes including impaired implantation, miscarriage, and restricted fetal growth (8–10). In animals, the HPT axis crosstalks with the hypothalamus–pituitary–gonadal (HPG) axis to regulate the seasonal breeding behavior (11, 12). However, the seasonal variation of thyroid function in reproductive-aged women has been rarely reported. Given the critical role of thyroid hormones on reproductive health, this study aims to evaluate the seasonal pattern of thyroid function changes in women of reproductive age by reviewing a large database spanning 7 years at a single hospital center in Northeast China.

## Materials and Methods

### Location and Definition of Seasons

Shenyang, the capital of Liaoning province in China, is located in latitude 41°11′–43°02′N and longitude 122°25′–123°48′E. This region is iodine adequate with a median urine iodine concentration (UIC) of 194 μg/L in adults (13). This inland city has a monsoon-influenced climate characterized by hot, humid summers and dry, cold winters. The monthly average temperatures range from -11.2°C in January to 24.6°C in July (14). Therefore, the distinct summer and winter climates in this region make Shenyang an appropriate location to investigate the effect of climatic variations on thyroid hormone levels in its population (15). In this study, “spring” was defined as being from March to May, “summer” from June to August, “autumn” from September to November, and “winter” from December to February.

### Subjects

**Figure 1:**
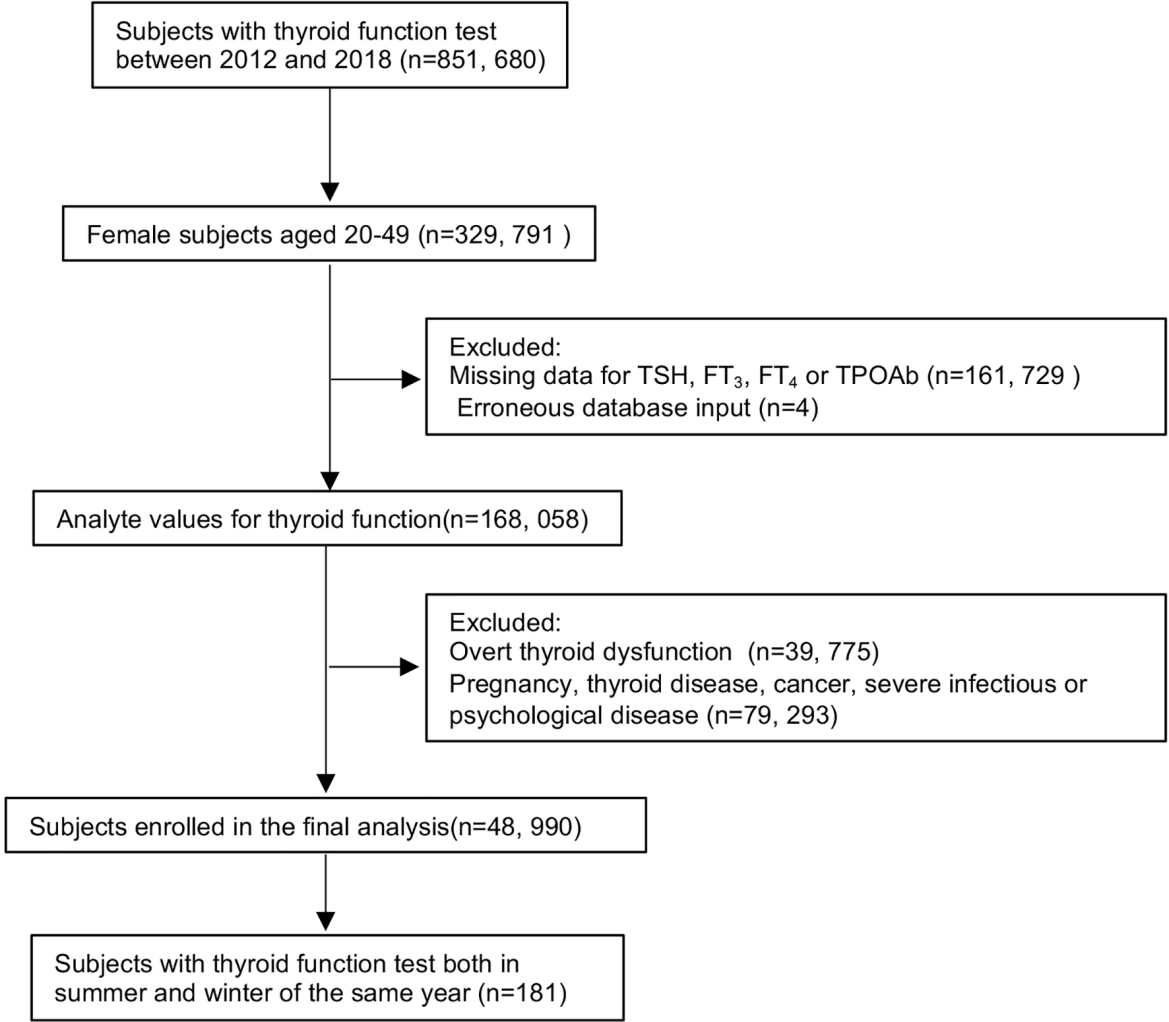
Flowchart of study participant inclusion and exclusion. Abbreviations: TSH, thyrotropin; FT_3_, free triiodothyronine; FT_4_, free thyroxine; TPOAb, thyroid peroxidase antibody.

**Figure 1** shows the flowchart of subject inclusion and exclusion. A total of 329,791 women aged 20–49 years who visited the outpatient or checkup center at the First Affiliated Hospital of China Medical University between January 2012 and December 2018 were included in this study. Subjects were excluded from the study if they were pregnant, had missing laboratory values [i.e., TSH, free triiodothyronine (FT_3_), free thyroxine (FT_4_), or thyroid peroxidase antibody (TPOAb) levels], had overt thyroid dysfunction (e.g., TSH <0.05 or >10 mIU/L, abnormal FT_3_ or FT_4_ levels), had thyroid disease, or erroneous database input (e.g., TSH or thyroid hormone levels exceeding the upper detection limit of the automated test kit). Subjects who had cancer or severe infectious or psychological disease were also excluded. In our final analyses, 48,990 subjects were included. In addition, 181 participants with thyroid function tests performed in both summer and winter of the same year were selected for longitudinal analysis.

### Measurement and Assessment of Thyroid Function

Thyroid function data were retrospectively collected from the hospital electronic database. At the time of the patient visit, blood samples were collected between 8:00 a.m. and 5:00 p.m. and analyzed on the same day of collection. Serum TSH, FT_3_, FT_4_, and TPOAb levels were assessed using the automated immunochemiluminescent assay (ICMA) kits (Abbott, IL, USA) at our central clinical laboratory. Inter-assay and intra-assay coefficients of variation were less than 5% for all analytes. Reference ranges based on the test kits for TSH, FT_3_, FT_4_, and TPOAb were 0.35–4.94 mIU/L, 2.63–5.70 pmol/L, 9.01–19.05 pmol/L, and 0–5.61 IU/ml, respectively. Thyroid function index [TSH index (TSHI)] is a parameter reflecting the feedback function to thyroid hormones and was calculated using the equation: TSHI = ln (TSH) + 0.1345 * FT_4_ (16). Higher values of TSHI indicates a lower central sensitivity to thyroid hormones (16). Subclinical thyroid hypothyroidism (SCH) was defined as TSH level >4.94 mIU/L with normal FT_3_ and FT_4_ levels, and subclinical hyperthyroidism (SHyper) was defined as TSH level <0.35 mIU/L with normal FT_3_ and FT_4_ levels (17).

This retrospective study was reviewed and approved by the Ethics Committee of China Medical University. The IRB approved to waive the documentation of informed consent because data used for this study were de-identified and did not involve confidential information and personal privacy.

### Statistical Analysis

The monthly, seasonal, and annual median levels of TSH, FT_3_, FT_4_, and TSHI were calculated. Distribution of continuous variables was measured using Kolmogorov–Smirnov test. All the variables were not normally distributed and presented as medians (interquartile ranges). Distribution ranges of thyroid parameters in our study subjects were defined as the 2.5th and 97.5th percentile (P_2.5_–P_97.5_) (18).

Prevalence of SCH and SHyper in different seasons was analyzed. The fluctuations of thyroid parameters were evaluated by the time series plot initially. The nonparametric Kruskal–Wallis test and analysis of means (ANOM) with transformed ranks were performed to compare the differences of thyroid function between seasons. Spearman correlation was used to investigate the relationship between thyroid parameters and age as a continuous variable. The Wilcoxon signed-rank test was performed to compare differences of thyroid function between summer and winter in the longitudinal analysis.

p < 0.05 was considered statistically significant for all results. Data collection and statistical analyses were performed using Excel version 2019, SPSS version 26.0, and R version 4.0.3 software.

## Results

The median age of the enrolled 48,990 participants was 39 years (interquartile range, 32–45 years). The median (interquartile range) of TSH, FT_3_, FT_4_, and TSHI was 1.79 (1.13–2.77) mIU/L, 4.21 (3.84–4.57) pmol/L, 13.29 (12.2–14.48) pmol/L, and 2.38 (1.92–2.81), respectively. The 2.5th and 97.5th percentile (P_2.5_–P_97.5_) of TSH, FT_4_, and FT_3_ was 0.22–6.56 mIU/L, 10.25–17.25 pmol/L, and 3.02–5.28 pmol/L, respectively. The prevalence of SCH and SHyper was 6.3% (3,086/48,990) and 4.2% (2,042/48,990), respectively.

### Seasonal Changes of Thyroid Function

**Table 1** shows the seasonal median of TSH, FT_3_, FT_4_, and TSHI. There were significant differences in TSH, FT_4_, and FT_3_ levels and TSHI among the four seasons (all p < 0.05 for Kruskal–Wallis test). The prevalence of SCH was lower in summer but higher in winter (5.6% *vs.* 7.0%, p < 0.05), with no significant difference in the prevalence of SHyper in the four seasons (p = 0.118).

**Table 1:** Comparisons of median TSH, FT_3_, FT_4_ and TSHI concentrations in four seasons.

	Spring (n = 12,637)	Summer (n = 12,658)	Autumn (n = 11,963)	Winter (n = 11,732)	Total (n = 48,990)	p (Kruskal–Wallis test)
TSH (mIU/L)	1.78 (1.15–2.78)	1.77 (1.11–2.72)	1.79 (1.14–2.75)	1.80 (1.13–2.83)	1.79 (1.13–2.77)	0.037
FT_4_ (pmol/L)	13.26 (12.12–14.50)	13.16 (12.10–14.35)	13.31 (12.24–14.46)	13.44 (12.37–14.61)	13.29 (12.2–14.48)	<0.001
FT_3_ (pmol/L)	4.22 (3.86–4.59)	4.13 (3.77–4.13)	4.24 (3.86–4.61)	4.25 (3.87–4.62)	4.21 (3.83–4.57)	<0.001
TSHI	2.38 (1.93–2.82)	2.36 (1.89–2.79)	2.38 (1.93–2.81)	2.41 (1.94–2.85)	2.38 (1.92–2.81)	<0.001

Data shown as median (interquartile range). TSH, thyrotropin; FT_3_, free triiodothyronine; FT_4_, free thyroxine; TSHI, TSH index.

**Figure 2:**
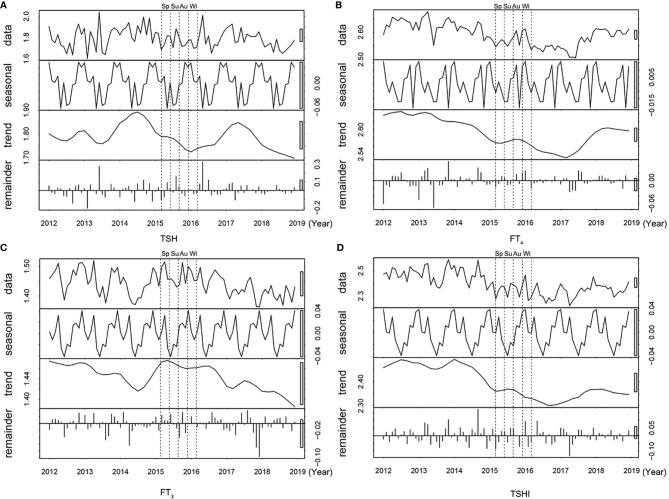
Time series plot of thyroid parameters over 7 years. The distribution (row 1), seasonal trend (row 2), time trend (row 3), and residuals (row 4) are shown for **(A)** TSH, **(B)** FT_4_, **(C)** FT_3_ levels, and **(D)** TSHI. Abbreviations: Sp, Spring; Su, Summer, Au, Autumn; Wi, Winter; TSH, thyrotropin; FT_3_, free triiodothyronine; FT_4_, free thyroxine; TSHI, TSH index.

**Figure 2** shows the time series plot of monthly variation in the median TSH, FT_3_, FT_4_, and TSHI over the 7 years. The levels of thyroid parameters fluctuated slightly each year, but all within the reference ranges (**Supplementary Table S1**). The median TSH, FT_3_, FT_4_, and TSHI all decreased during the summer but increased during the winter significantly (all p < 0.05 for ANOM test). Among participants with positive or negative TPOAb, seasonal variations of thyroid function were consistently similar with a peak in winter and trough in summer (all p < 0.001 for ANOM test) (**Supplementary Figure S1**).

### Relationship Between Thyroid Parameters and Age

The distribution of TSH, FT_4_, FT_3_, and TSHI based on age groups is listed in **Table 2**. Both the 2.5th and 97.5th percentiles of TSH were slightly higher in women aged 40–49 years than women aged 20–29 years. Spearman correlation analysis showed that TSH (r = 0.044, p < 0.001) and TSHI (r = 0.025, p < 0.001) were positively associated with age, while FT_3_ (r = -0.073, p < 0.001) and FT_4_ (r = -0.059, p < 0.001) were negatively associated with age. The associations of thyroid parameters with age were similar between subjects with positive TPOAb and those with negative TPOAb (all p < 0.001 for Spearman’s correlation) (**Supplementary Table S2**).

**Table 2:** Distribution of TSH, FT_4_, and FT_3_ by age group.

Age (years)	TSH (mIU/L)		FT_4_ (pmol/L)		FT_3_ (pmol/L)	
	Median	P_2.5_–P_97.5_	Median	P_2.5_–P_97.5_	Median	P_2.5_–P_97.5_
20–29 (n = 8, 836)	1.74	0.20–6.53	13.52	10.22–17.45	4.29	3.00–5.38
31–39 (n = 16, 166)	1.72	0.21–6.33	13.31	10.31–17.22	4.22	3.05–5.28
40–49 (n = 23, 988)	1.85	0.24–6.75	13.19	10.22–17.21	4.17	3.01–5.25
Total (n = 48, 990)	1.79	0.22–6.56	13.29	10.25–17.25	4.21	3.02–5.28

TSH, thyrotropin; FT_3_, free triiodothyronine; FT_4_, free thyroxine.

### Longitudinal Analysis for the Seasonal Variation of Thyroid Function

**Figure 3:**
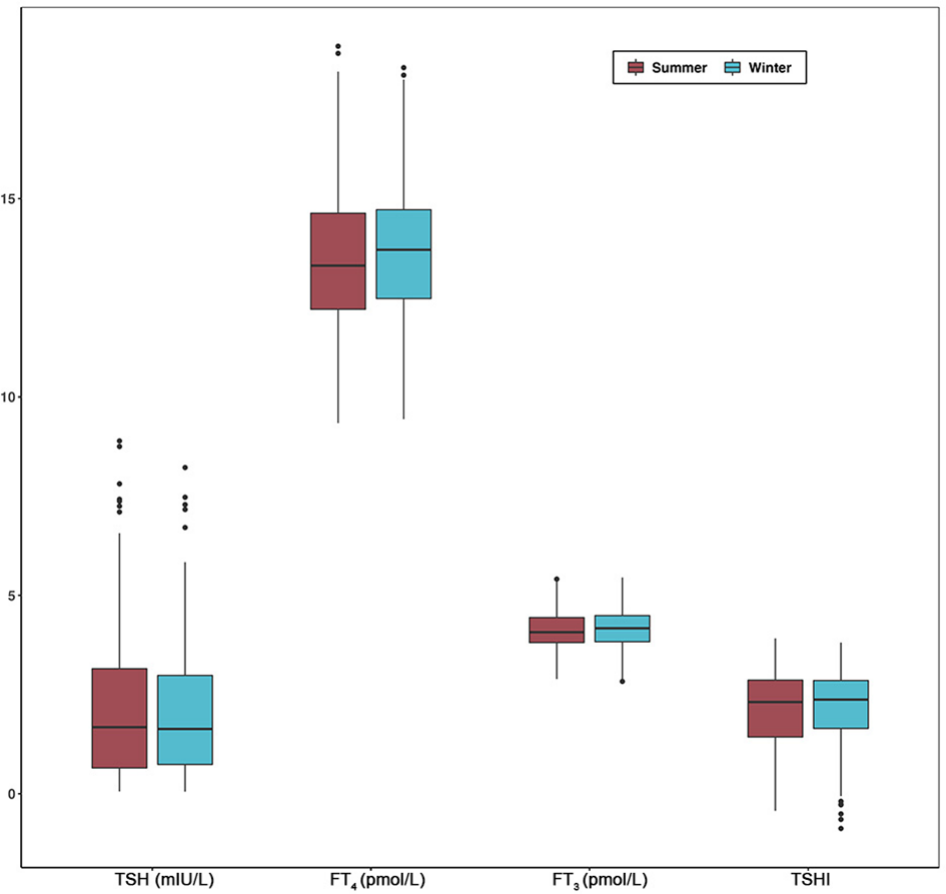
Comparisons of TSH, FT_4_, FT_3_, and TSHI between summer and winter in longitudinal analysis. Box plot indicates upper and lower quartiles (box) with median (central line). Whiskers indicate upper and lower extremes. Abbreviations: TSH, thyrotropin; FT_3_, free triiodothyronine; FT_4_, free thyroxine; TSHI, TSH index; TPOAb, thyroid peroxidase antibody.

Since seasonal differences of thyroid parameters were most significant between summer and winter, we selected 181 subjects with thyroid function tests performed in both seasons of the same year. The median FT_3_, FT_4_, and TSHI were slightly higher in the winter compared to summer, whereas the median TSH concentrations were lower in winter compared to summer (**Figure 3**). However, there were no significant differences in the thyroid parameters between the two seasons in the Wilcoxon signed-rank test (**Supplementary Table S3**).

## Discussion

This retrospective single-center study showed that thyroid hormone levels and central sensitivity to thyroid hormones are influenced by age and seasonal factors among 48,990 women of reproductive age. Their TSH and peripheral thyroid hormone levels peaked during winter and declined to a trough during summer. Sensitivity to thyroid hormones (reflected by TSHI), on the other hand, increased in summer and decreased in winter. In addition, TSH and TSHI showed a positive correlation with age, while peripheral thyroid hormone levels were negatively associated with age. Subgroup analysis suggests that our findings were independent of thyroid autoantibodies. In the longitudinal analysis of 181 subjects, no significant differences were detected in the thyroid parameters between summer and winter.

Consistent with our observations, previous studies in healthy adults have shown that TSH levels decreases in summer–spring, while it increases in autumn–winter (6, 7, 15, 19–25). The seasonal variation of TSH between winter and summer reached an absolute difference of more than 10%–15% in a multinational study of Belgian, British, and Japanese residents (21). For healthy subjects, the seasonal fluctuation of TSH levels may be an adaptation of energy intake and metabolic rate during different climates throughout the year. However, this may not apply for subjects with thyroid disease. A retrospective study found that in subjects with SCH, more patients reverted to euthyroid status during summer–fall season than winter–spring season (64.4% *vs.* 52.7%) (6). Similarly, we also observed an increased prevalence of SCH in reproductive-aged women during winter. However, for patients with thyroid disease, there is no conclusive evidence suggesting that thyroid medication should be adjusted according to different seasons.

Our study extends previous work on this topic in several ways. Firstly, using the big data approach, we presented the seasonal pattern of thyroid function in women at reproductive age for the first time. Thyroid function and reproduction are closely linked. Adequate thyroid hormones are vital for the maturation and function of ovarian, uterine, and placental tissue (26), which are important for optimal pregnancy outcomes and fetal development (9). Fetuses suffering from intrauterine thyroid dysfunction due to maternal thyroid dysfunction tend to have increased risk of thyroid cancer and impaired physical and cognitive function (27, 28). Therefore, we presume that optimal thyroid status at the time of conception may help improve fetal–maternal health outcomes. Secondly, we introduced the calculation of TSHI in our analysis. The evaluation of the HPT axis feedback function by TSHI composite index is more accurate compared to the single parameter of TSH in previous studies (7, 21, 23). Our analysis supports the finding that central sensitivity to thyroid hormones was influenced by seasonal factors and age.

However, it should be noted that although the current data showed a significant seasonal pattern of TSH, the median TSH levels in the four seasons were all within the recommended TSH range for pregnancy during the first trimester (~2.5 mIU/L) (29). Despite increased prevalence of thyroid dysfunction in winter compared to summer, the difference was minimal (7.0% *vs.* 5.6%) and its impact remains to be clarified. Hence, the statistical difference in this study should not be interpreted as clinical significance.

The combination of experimental and clinical studies may be helpful in elucidating the mechanism of seasonal impacts on thyroid physiology. Firstly, temperature variation is a critical stimulus to the central regulation of HPT axis (30). Studies on residence in the Antarctica suggest that prolonged cold exposure was associated with increased TSH and T_3_ levels, a phenomenon called “Polar T3 Syndrome” (31). Similarly, our study also observed increased TSH and FT_3_ levels during winter compared to summer. Besides TSH and thyroid hormone levels, our study showed that the HPT set point (reflected by TSHI) also changed in winter. This may represent a form of type 2 thyroid allostasis in which production of active thyroid hormones will be upregulated in environments of increased energy demand such as pregnancy and exposure to cold weather (32). Secondly, light or photoperiod was also associated with seasonal fluctuations of thyroid function. Precise control of thyroid activity is regulated by the opposing actions of deiodinases (Dio). Studies showed that melatonin, a hormone produced and released within the pineal gland in response to light signals, inhibits the expression of Dio2 while triggering Dio3 expression (33). In addition, the alteration in thyroid hormone regulation could also be a part of metabolic adaptation to seasonal climate changes. Tendler et al. (34) analyzed an Israeli medical record of 46 million person-years, and they found that most pituitary hormone levels peaked in summer, while their effector hormones peaked in winter–spring. It suggests that many feedback systems taking part in growth, metabolism, and reproduction axes in humans have seasonal set points (34). Further investigations are warranted to explore the main influencing factors participating in the thyroid adaptation to different seasons.

There are four main limitations in this study. Firstly, we draw our main conclusions from a cross-sectional study. However, the possibility of causal correlations should not be completely dismissed. To compensate for this, we performed a longitudinal analysis in a small group of 181 subjects who were followed in both summer and winter. Although we did not find significant differences in the longitudinal study as we have found in the big data analysis, it is possible that the sample size of the longitudinal study was too small to reach statistical power. Secondly, despite the significant seasonal temperature variation in Shenyang, the widespread use of improved indoor heating and cooling technologies in the city may have dampened the impact of external temperature changes on thyroid function. Thirdly, although our subjects resided in an iodine-adequate area (13), seasonal variations in iodine intake may influence the synthesis and secretion of thyroid hormones (35). To determine the change of peripheral metabolism of thyroid hormones throughout the year, it is useful to calculate the thyroid’s secretory capacity (SPINA-GT) and the sum activity of peripheral step-up deiodinases (SPINA-GD) (36). However, we were unable to calculate these two parameters because the equation requires total thyroid hormone levels and thyroxine-binding globulin (TBG) levels, which were absent in our database. Finally, this analysis enrolled women of reproductive age at a single hospital center. Apart from age, thyroid function can also be influenced by other variates such as iodine intake and ethnicity (37), which we were unable to analyze in this retrospective single-center study. Therefore, prospective observational studies are necessary to further corroborate the generalizability of our conclusions.

In conclusion, we have shown that the parameters of thyroid function (TSH, FT_4_, and FT_3_) in women of reproductive age are higher in winter and lower in summer, while central sensitivity to thyroid hormones increased in summer and decreased in winter. Although we cannot determine the clinical significance of this change based on a cross-sectional study, our study indicates that it is valuable to further investigate whether the thyroid function differences between seasons have impacts on reproductive health. More longitudinal evidence is warranted to elucidate whether the circannual variability of thyroid function impacts fetal–maternal outcomes and whether prescription of thyroid medication should be adjusted during different seasons for women planning to conceive.

## Data Availability Statement

The original contributions presented in the study are included in the article/**Supplementary Material**. Further inquiries can be directed to the corresponding authors.

## Ethics Statement

The studies involving human participants were reviewed and approved by Ethics Committee of China Medical University. Written informed consent for participation was not required for this study in accordance with the national legislation and the institutional requirements.

## Author Contributions

JF, GZ, RG, PX, and JL contributed to the acquisition, analysis, and interpretation of data. JF and GZ drafted the manuscript. YL and HG designed and supervised the study and helped revise the manuscript. JF and GZ contributed equally to this work as first authors. YL and HG contributed equally as corresponding authors. All authors contributed to the article and approved the submitted version.

## Funding

This study was funded by the National Natural Science Foundation of China (Grant No. 81870538).

## Conflict of Interest

The authors declare that the research was conducted in the absence of any commercial or financial relationships that could be construed as a potential conflict of interest.

## Publisher’s Note

All claims expressed in this article are solely those of the authors and do not necessarily represent those of their affiliated organizations, or those of the publisher, the editors and the reviewers. Any product that may be evaluated in this article, or claim that may be made by its manufacturer, is not guaranteed or endorsed by the publisher.
