# Supplementary material for: Seasonal Changes of Thyroid Function Parameters in Women of Reproductive Age Between 2012 and 2018: A Retrospective, Observational, Single-Center Study
Source: Front Endocrinol (Lausanne). 2021 Sep 2;12:719225. doi: 10.3389/fendo.2021.719225 (PMC8443767; doi:10.3389/fendo.2021.719225)
Supplement: Supplementary file 1 [file DataSheet_1.docx]

Supplementary Material

Supplementary Table 1. Thyroid function parameters by year. Data shown as median (interquartile range).

| Year | TSH (mIU/L) | FT_4_ (pmol/L) | FT_3_ (pmol/L) | TSHI |
| --- | --- | --- | --- | --- |
| 2012 (n=4,558) | 1.78 (1.12, 2.78) | 13.96 (12.82, 15.20) | 4.34 (3.95, 4.75) | 2.48 (2.00, 2.90) |
| 2013 (n=4,362) | 1.81 (1.12, 2.84) | 13.86 (12.67, 15.12) | 4.29 (3.85, 4.69) | 2.47 (1.99, 2.92) |
| 2014 (n=6,685) | 1.86 (1.18, 2.88) | 13.58 (12.46, 14.77) | 4.14 (3.77, 4.50) | 2.46 (2.00, 2.89) |
| 2015 (n=7,339) | 1.78 (1.14, 2.75) | 13.10 (12.09, 14.19) | 4.34 (3.94, 4.72) | 2.35 (1.91, 2.77) |
| 2016 (n=7,716) | 1.77 (1.29, 2.77) | 12.80 (11.79, 13.88) | 4.31 (3.95, 4.65) | 2.31 (1.84, 2.74) |
| 2017 (n=8,165) | 1.83 (1.18, 2.81) | 12.81 (11.77, 13.92) | 4.11 (3.77, 4.43) | 2.34 (1.90, 2.76) |
| 2018 (n=10,165) | 1.71 (1.08, 2.66) | 13.51 (12.47, 14.62) | 4.10 (3.76, 4.42) | 2.36 (1.90, 2.80) |

Abbreviations: TSH, thyrotropin; FT_3_, free triiodothyronine; FT_4_, free thyroxine; TSHI, TSH index.

Supplementary Table 2. Spearman’s correlation analysis between age and thyroid function as continuous variables among subjects with positive or negative thyroid peroxidase antibodies (TPOAb).

|  | Age (years) | |
| --- | --- | --- |
|  | r | *P* |
| TPOAb(-) group (n=32,936) | | |
| TSH(mIU/L) | 0.039 | <0.001 |
| FT_4_(pmol/L) | -0.045 | <0.001 |
| FT_3_(pmol/L) | -0.066 | <0.001 |
| TSHI | 0.023 | <0.001 |
| TPOAb(+) groups (n=16,054) | | |
| TSH | 0.080 | <0.001 |
| FT_4_ | -0.093 | <0.001 |
| FT_3_ | -0.088 | <0.001 |
| TSHI | 0.053 | <0.001 |

Abbreviations: TSH, thyrotropin; FT_3_, free triiodothyronine; FT_4_, free thyroxine; TSHI, TSH index; TPOAb: thyroid peroxidase antibodies.

Supplementary table 3. Comparisons of TSH, FT_4_, FT_3_ and TSHI between summer and winter in longitudinal analysis in 181 subjects.

|  | Summer (Mean±SEM) | Winter (Mean±SEM) | *P*  (Wilcoxon signed-rank test ) |
| --- | --- | --- | --- |
| TSH (mIU/L) | 2.13±0.14 | 2.05±0.12 | 0.979 |
| FT_4_ (pmol/L) | 13.58±0.14 | 13.71±0.14 | 0.322 |
| FT_3_ (pmol/L) | 4.11±0.04 | 4.18±0.04 | 0.099 |
| TSHI | 2.06±0.08 | 2.17±0.07 | 0.287 |

Abbreviations: TSH, thyrotropin; FT_3_, free triiodothyronine; FT_4_, free thyroxine; TSHI, TSH index; TPOAb: thyroid peroxidase antibodies; SEM, standard error of the mean.


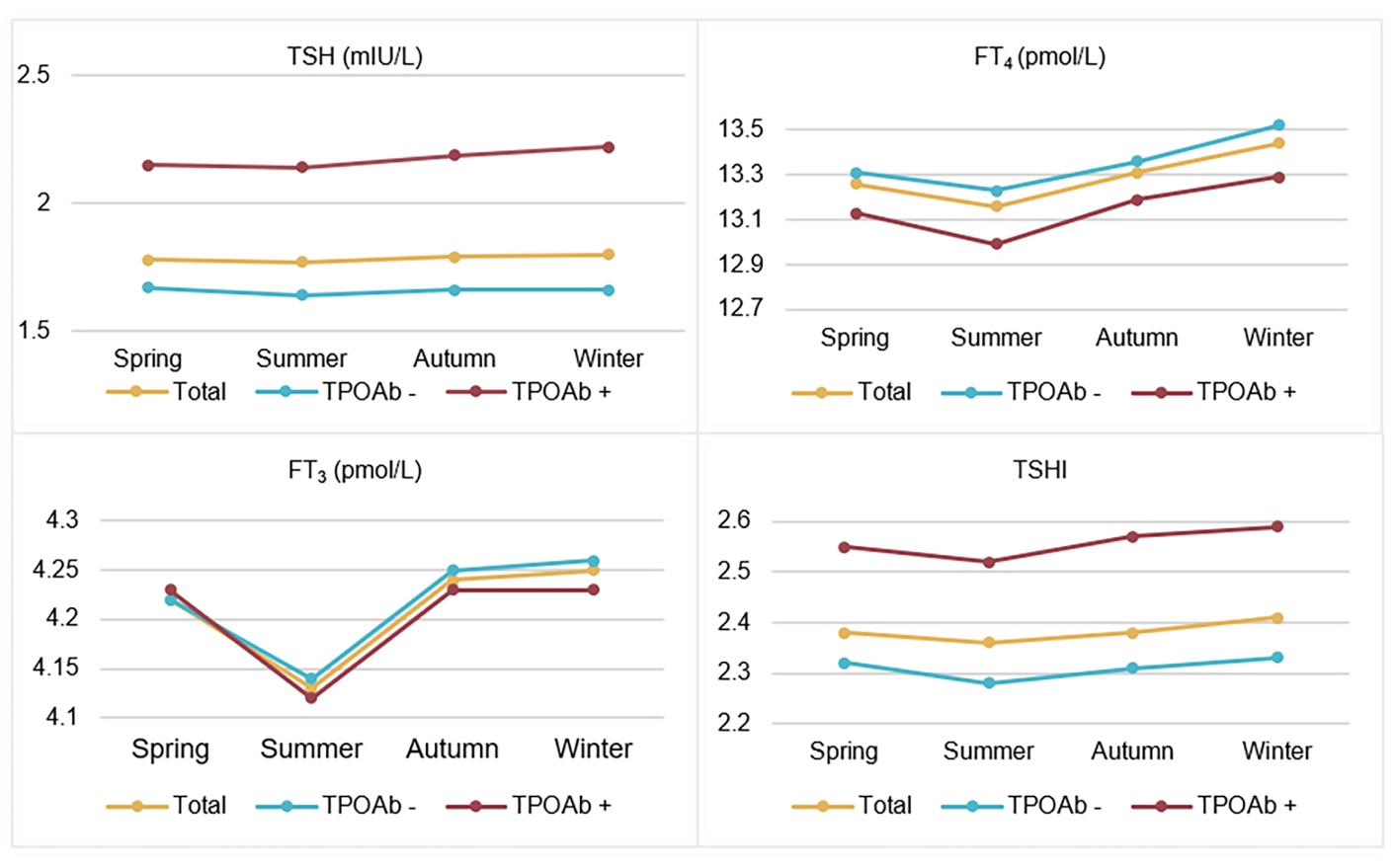


Supplementary Figure 1. Comparisons of thyroid function between four seasons among subjects with negative or positive TPOAb. Abbreviations: TSH, thyrotropin; FT_3_, free triiodothyronine; FT_4_, free thyroxine; TSHI, TSH index; TPOAb: thyroid peroxidase antibodies.
